# Cyclin-dependent kinase 4/6 inhibitors in granulosa cell tumors and their combination with hormonal therapy: a scoping review

**DOI:** 10.3389/fonc.2026.1844395

**Published:** 2026-06-26

**Authors:** Renata Pacholczak-Madej, Radosław Łupkowski, Karolina Górniak, Mirosława Puskulluoglu

**Affiliations:** 1 Department of Gynecological Oncology, Maria Sklodowska-Curie National Research Institute of Oncology, Krakow, Poland; 2 Department of Anatomy, Jagiellonian University, Medical College, Krakow, Poland; 3 Department of Clinical Oncology, Maria Sklodowska-Curie National Research Institute of Oncology, Krakow, Poland

**Keywords:** adult granulosa cell tumor, CDK4/6 inhibitors, cell cycle, cyclin-dependent kinases, endocrine therapy

## Abstract

**Background:**

Adult granulosa cell tumor (aGCT) is a rare ovarian malignancy with frequent late recurrences and limited systemic treatment options. Emerging evidence suggests that dysregulation of the cyclin D–cyclin-dependent kinase (CDK) 4/6–retinoblastoma (Rb) axis contributes to aGCT biology, while hormone receptor expression supports the potential role of endocrine therapy. We performed a scoping review to map available evidence on CDK4/6 inhibition in aGCT.

**Methods:**

A literature search was conducted in PubMed/MEDLINE, Embase and Web of Science, complemented by searches of trial registries, conference abstracts and citation searching. Eligible reports included translational, preclinical and clinical studies addressing CDK4/6 pathway dysregulation or CDK4/6 inhibition in aGCT. Evidence on endocrine therapy was synthesized narratively to contextualize combination strategies.

**Results:**

Twenty-three reports were included. Translational and genomic studies showed recurrent abnormalities affecting cell-cycle regulation, including alterations in CDK inhibitors, Rb-associated signaling and other proliferation-related pathways. Preclinical studies demonstrated that CDK4/6 inhibition, particularly with abemaciclib, reduced cell viability and tumor growth in GCT models. Clinical evidence was limited to small retrospective case series, which suggested clinically meaningful disease control in a subset of patients with recurrent disease. An ongoing phase II trial is expected to provide the first systematic evaluation of this therapeutic strategy.

**Conclusions:**

Available evidence supports CDK4/6 inhibition as a biologically plausible therapeutic strategy in aGCT. Although current clinical data remain limited, the combination of CDK4/6 inhibitors with endocrine therapy appears promising and warrants prospective evaluation.

## 1 Introduction

Adult granulosa cell tumor (aGCT) is a rare ovarian malignancy, accounting for approximately 2–5% of ovarian cancers and representing the most common subtype of sex cord-stromal tumors. The disease typically exhibits an indolent course, with most patients diagnosed at an early stage and achieving 10-year survival rates exceeding 80% (1). Despite this favorable initial prognosis, aGCT is characterized by a marked risk of late relapse—nearly half of recurrences in the MITO study occurred more than five years after diagnosis—highlighting the need for long-term surveillance (2). In recurrent disease, treatment is individualized and may include repeat surgery, endocrine therapy and systemic approaches such as chemotherapy or antiangiogenic therapy. When feasible, platinum-based chemotherapy, often combined with bevacizumab, a vascular endothelial growth factor (VEGF) inhibitor, remains a commonly used option; however, responses are frequently not durable (3, 4).

A defining molecular feature of aGCT is the FOXL2 c.402C>G (p.C134W) mutation, present in nearly all tumors (5). FOXL2 (forkhead box L2) is a transcription factor involved in granulosa cell differentiation, ovarian maintenance and steroidogenesis. The recurrent FOXL2 p.C134W alteration is considered a gain-of-function mutation that disrupts transcriptional regulation. It promotes overexpression of cyclin D isoforms such as cyclin D2 and enhances signaling through the cyclin-D–cyclin-dependent kinases (CDK) 4/6–retinoblastoma (Rb) pathway, driving unchecked G1–S cell-cycle progression. The FOXL2 mutation also induces aromatase expression. Increased aromatase expression may enhance local estrogen production and estrogen receptor signaling in aGCT. Upon ligand binding, estrogen receptors regulate transcription of proliferation-associated genes, including cyclin D isoforms, thereby promoting activation of the cyclin D–CDK4/6–Rb pathway and G1/S cell-cycle progression (6, 7). Given that aGCT frequently expresses estrogen (ER) and progesterone receptors (PR) detected by immunohistochemistry in 66% and 98%, respectively (8), endocrine therapies may attenuate hormone-mediated growth and potentially amplify the antiproliferative effects of CDK4/6 inhibition (9).

Taken together, these molecular insights and emerging clinical observations underscore the need for a systematic mapping of the available translational, preclinical and clinical evidence. This scoping review aims to synthesize current knowledge on CDK4/6 inhibition in aGCT and to evaluate the therapeutic rationale for combining these agents with endocrine strategies—particularly aromatase inhibitors and fulvestrant—as a basis for future clinical investigation. The following research question was formulated: What translational, preclinical and clinical evidence is currently available regarding the use of CDK4/6 inhibitors in aGCT?

## 2 Methods

### 2.1 Protocol and reporting

The protocol for this scoping review was developed in accordance with the Preferred Reporting Items for Systematic Reviews and Meta-Analyses for Scoping Reviews (PRISMA-ScR) guidelines [10], with details on their implementation provided in **Supplementary Table 1**. The draft protocol was reviewed and refined by all members of the research team to ensure methodological rigor and alignment with the study objectives. The finalized protocol was registered prospectively on the Open Science Framework (OSF) on *15 Dec 2025* (registration link: https://osf.io/nqhxw).

### 2.2 Eligibility criteria

This scoping review was structured according to the Population–Concept–Context framework. The Population comprised patients with aGCT and corresponding *in vitro* and *in vivo* models of aGCT. The Concept was CDK4/6 inhibition, including studies assessing CDK4/6 pathway dysregulation, pharmacologic CDK4/6 inhibitors and related therapeutic strategies. The Context included translational, preclinical and clinical settings in which CDK4/6 inhibition was investigated in aGCT. We included peer-reviewed clinical studies, case reports, case series, conference abstracts, translational research and preclinical studies examining CDK4/6 inhibition and CDK4/6 pathway dysregulation in aGCT. No restrictions were applied regarding study design or publication type, given the expected scarcity of data. We limited the search to English-language publications to ensure accurate interpretation of scientific content. No date limits were imposed to allow for the identification of all relevant studies from the database’s inception to the final search date.

### 2.3 Literature search

A comprehensive literature search was conducted in PubMed/MEDLINE, Embase and Web of Science databases using the following search strategy: (“granulosa cell tumor” OR “adult granulosa cell tumor” OR “sex cord stromal tumor”) AND (“cyclin-dependent kinase” OR CDK4 OR CDK6 OR CCND1 OR “cell cycle”) by two independent researchers (RPM and RŁ). The search strategy was adapted for Embase to improve specificity and reduce retrieval of irrelevant records while preserving the core search concepts (“granulosa cell tumor” OR “adult granulosa cell tumor”) AND (“cyclin-dependent kinase” OR CDK4 OR CDK6).

To ensure a comprehensive overview of emerging data, we also conducted a hand search of conference abstracts from the American Society for Clinical Oncology *(*ASCO), the European Society for Medical Oncology (ESMO), the ESMO Gynecological Cancers and the European Society for Gynecological Oncology (ESGO) Congresses for the years 2016 through February 2026. Additionally, we reviewed records from major clinical trial registries, including ClinicalTrials.gov and the EU Clinical Trials Register. Reference lists of included studies were also screened to identify any additional relevant publications. The search strategy was intentionally limited to studies evaluating CDK4/6 inhibitors in aGCT. Evidence regarding potential endocrine combination partners, including aromatase inhibitors and fulvestrant, was synthesized narratively based on biological rationale and information extracted from included studies as well as relevant background literature identified through hand-searching of reference lists. No independent systematic search dedicated specifically to endocrine therapy combinations was performed.

The final search was conducted on 1 February 2026.

### 2.4 Data charting

**Figure 1:**
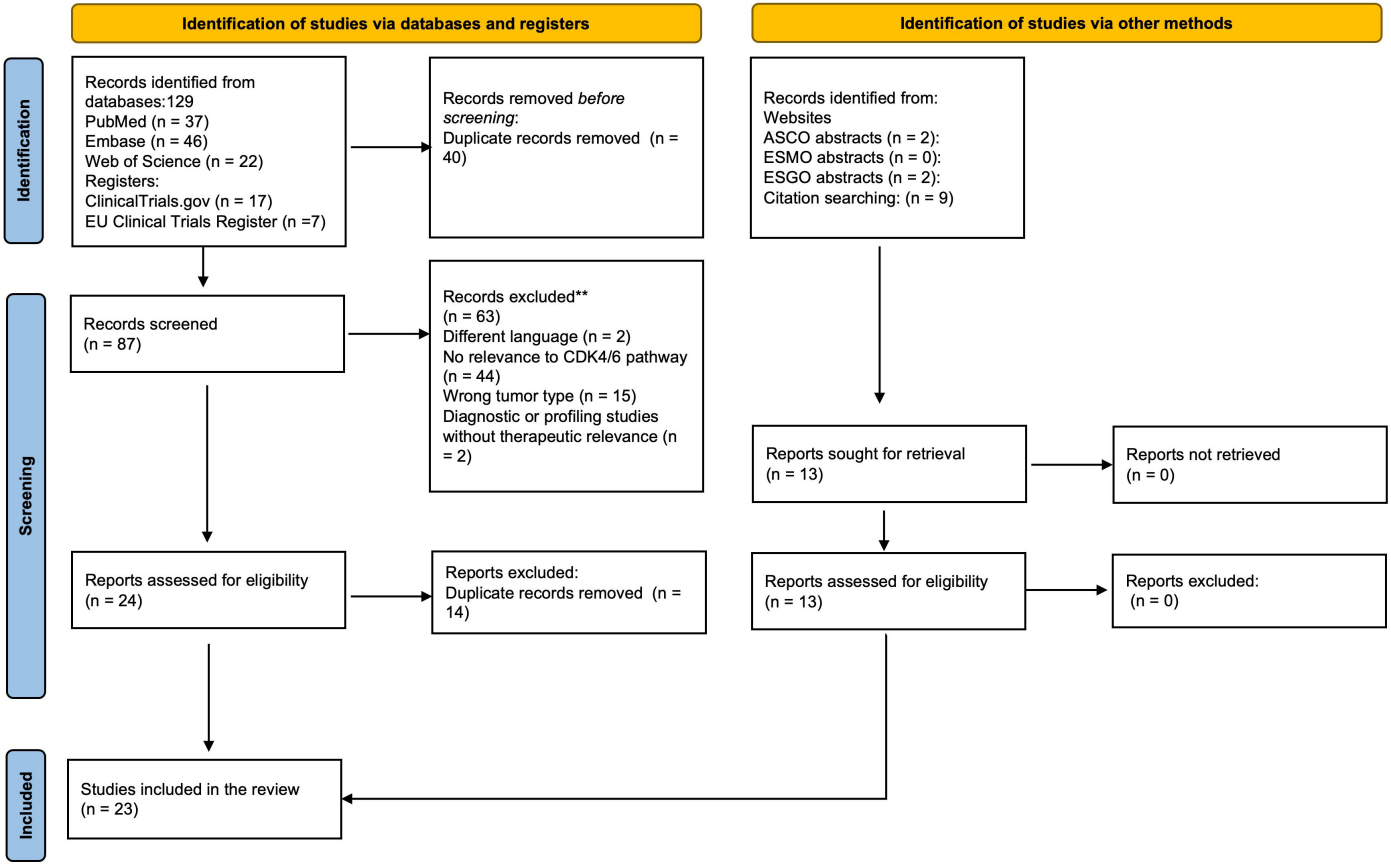
PRISMA 2020 flow diagram of study selection for the scoping review. ASCO, American Society of Clinical Oncology; ESGO, European Society of Gynaecological Oncology; ESMO, European Society for Medical Oncology; EU, European Union; PRISMA, Preferred Reporting Items for Systematic Reviews and Meta-Analyses.

The overall procedure for study identification, screening, eligibility assessment and final inclusion is depicted in a PRISMA 2020 flow diagram, adapted in accordance with methodological guidance for scoping reviews (**Figure 1**) (10). When multiple reports referred to the same study (e.g., a conference abstract and a subsequent full publication or a preprint), they were linked as a single study in the synthesis.

### 2.5 Synthesis of results

Evidence from the included studies was synthesized using descriptive and thematic approaches aligned with established scoping review methodology. Given the marked variability in study designs, interventions and clinical settings, a quantitative meta-analysis was not performed. The results were synthesized and presented narratively and systematically grouped into major thematic categories.

## 3 Translational and preclinical evidence supporting cell-cycle targeting in granulosa cell tumors

**Figure 2:**
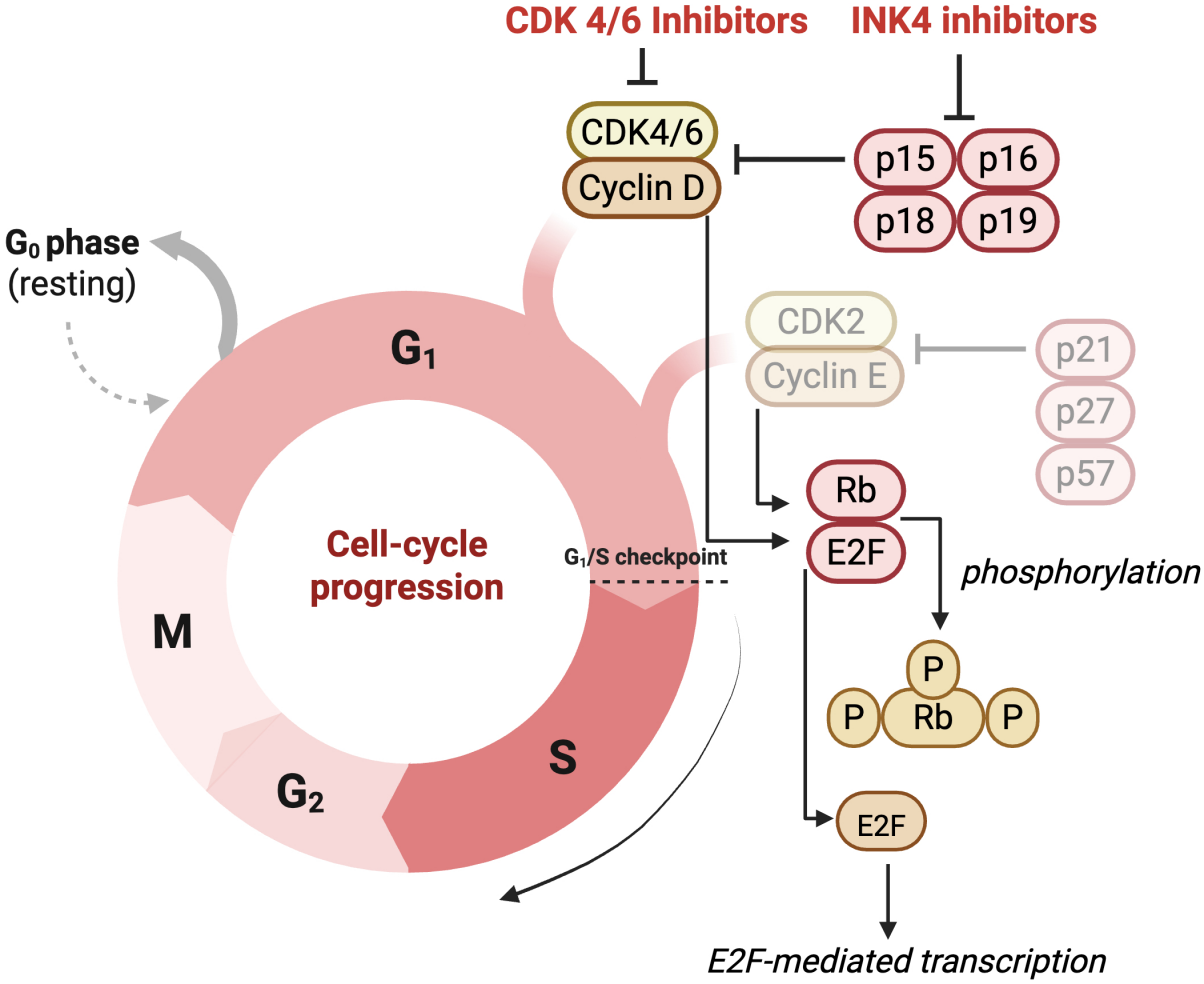
Regulation of the cyclin D–CDK4/6–Rb pathway and mechanism of CDK4/6 inhibition. The G1/S transition is regulated by cyclin D–CDK4/6 complexes, which phosphorylate the retinoblastoma (Rb) protein, resulting in the release of E2F transcription factors and promotion of cell-cycle progression. Endogenous CDK inhibitors, including members of the INK4 family (p15, p16, p18 and p19) negatively regulate CDK4/6 activity. Pharmacologic CDK4/6 inhibitors suppress Rb phosphorylation and prevent progression through the G1/S checkpoint. Inhibitory molecules and interactions are highlighted in red, whereas stimulatory pathways are depicted in yellow/orange. *CDK, cyclin-dependent kinase; E2F, E2F transcription factor; G0, quiescent phase; G1, gap 1 phase; G2, gap 2 phase; M, mitosis; P, phosphorylation; Rb, retinoblastoma protein.*.

Cell-cycle progression is tightly regulated by interactions between cyclins, CDKs and endogenous CDK inhibitors. Transition from G1 to S phase is primarily controlled by cyclin D–CDK4/6 complexes, which phosphorylate the Rb protein, resulting in the release of E2F transcription factors and initiation of DNA synthesis. This process is negatively regulated by endogenous CDK inhibitors, including members of the INK4 family (p15, p16, p18, p19) and CDK-interacting protein/kinase-inhibitory protein (CIP/KIP) (p21, p27 and p57). Dysregulation of this pathway promotes uncontrolled proliferation and represents a common oncogenic mechanism across multiple malignancies (11, 12) (**Figure 2**).

Genomic studies consistently demonstrate dysregulation of cell-cycle regulatory pathways in aGCT. Analyses of paired primary and recurrent tumors indicate enrichment of cell-cycle–related alterations in recurrent disease, including mutations in Tumor Protein 53 (TP53), Mediator Complex Subunit 12 (MED12), as well as Telomerase Reverse Transcriptase (TERT) promoter alterations (13, 14). Large molecular profiling studies further support the importance of cell-cycle dysregulation, identifying recurrent alterations in genes involved in CDK signaling, including deletions of CDKN2A/CDKN2B in approximately 10% of tumors (15, 16). Although the cyclin D–CDK4/6–Rb pathway appears central to aGCT biology, additional signaling pathways may also contribute to tumor proliferation. Pharmacologic inhibition of the c-Jun N-terminal kinase (JNK) pathway in the COV434 granulosa tumor cell line. While the model not fully representative of aGCT, resulted in dose-dependent growth arrest, accumulation of cells in the G2/M phase and induction of apoptosis, suggesting that JNK signaling may represent another regulator of cell-cycle progression in these tumors (17).

Early molecular analyses suggested that disruption of endogenous CDK inhibitors contributes to granulosa cell tumorigenesis. Loss of INK4A or INK4B expression was detected in more than half of adult tumors examined in an early cohort. INK4 proteins normally suppress CDK4/6 activity by preventing their association with cyclin D and limiting Rb phosphorylation, thereby restraining G1–S cell-cycle progression. Loss of INK4 expression, therefore, removes a critical regulatory checkpoint and may promote CDK4/6 activation and tumor cell proliferation (18). Additional support for dysregulation of this pathway comes from immunohistochemical studies demonstrating increased stromal expression of p16INK4A in aGCT compared with normal ovarian tissue and benign sex cord-stromal tumors, with even higher expression observed in recurrent tumors, suggesting a potential role of the tumor microenvironment in disease progression (19).

Experimental models further reinforce the importance of Rb-dependent cell-cycle control. In a mouse model combining granulosa cell–specific deletion of Rb with inhibin-α deficiency, ovarian tumors displayed increased proliferation and reduced expression of the CDK inhibitor p27, indicating that disruption of Rb signaling promotes tumor growth and aggressiveness (20). Similarly, studies of transcriptional regulators have shown that Runt-related transcription factor 3 (RUNX3) promotes proliferation and tumor progression in granulosa cell tumor models by increasing cyclin D2 expression and reducing p27 levels, thereby facilitating CDK4/6 activation and G1 cell-cycle progression (21).

Direct therapeutic support for this biological rationale has been provided by preclinical studies evaluating pharmacologic CDK4/6 inhibition. In KGN GCT cells and patient-derived organoid models, the CDK4/6 inhibitor abemaciclib demonstrated potent antiproliferative activity, while treatment of xenograft mouse models significantly reduced tumor volume and tumor mass compared with controls (22, 23).

Collectively, these findings indicate that dysregulation of the cyclin D–CDK4/6–Rb axis is a recurrent biological feature of aGCT and provide a strong biological rationale for combined endocrine and CDK4/6-targeted therapeutic strategies, as discussed in the following section.

## 4 Evidence for endocrine therapy in recurrent adult granulosa cell tumors

Because aGCTs are frequently hormonally active tumors characterized by ER and PR expression, multiple endocrine therapeutic approaches have been explored in recurrent disease. Several endocrine approaches have been used in clinical practice, including synthetic progestins, selective estrogen receptor modulators, aromatase inhibitors and gonadotropin-releasing hormone agonists (GnRHa) (24). In a retrospective cohort of patients with measurable recurrent or residual disease, hormone therapy achieved an 18% objective response rate (ORR) and 64% stable disease (SD) (24). A systematic review of 19 studies (31 patients) reported a pooled ORR of 71% (95% confidence interval [CI] 52–85%), with 25.8% complete responses (CR) and 45.2% partial responses (PR) (9). These findings should be interpreted cautiously, given the small sample size and the nature of the included reports. Aromatase inhibitors produced responses in all reported treatment courses (9/9), whereas tamoxifen showed no responses in three cases (9). Prospective data are scarce, but the PARAGON phase II trial of anastrozole in recurrent or metastatic ER- and/or PR-positive aGCT showed a 78.9% clinical benefit rate at 12 weeks, 10.5% PR and a median progression-free survival (PFS) of 8.6 months (25). Additional case reports and small series support endocrine activity: letrozole induced radiologic shrinkage in a heavily pretreated recurrent case, avoiding further surgery (26); combined leuprolide and tamoxifen normalized inhibin B and maintained control for two years after multiple prior therapies (27); alternating megestrol acetate and tamoxifen reduced inhibin levels and prolonged disease stabilization in recurrent disease (28). In a series with over 20 years of follow-up, patients with stage IV recurrent tumors achieved prolonged survival with multiple treatment lines, including tamoxifen, letrozole and tamoxifen–megestrol combinations, which contributed to sustained disease stability (29). Indirect supportive evidence also comes from ER-targeting agents such as fulvestrant, which have achieved disease stabilization in ER-positive low-grade gynecologic malignancies, including sex cord–stromal tumors (30).

**Figure 3:**
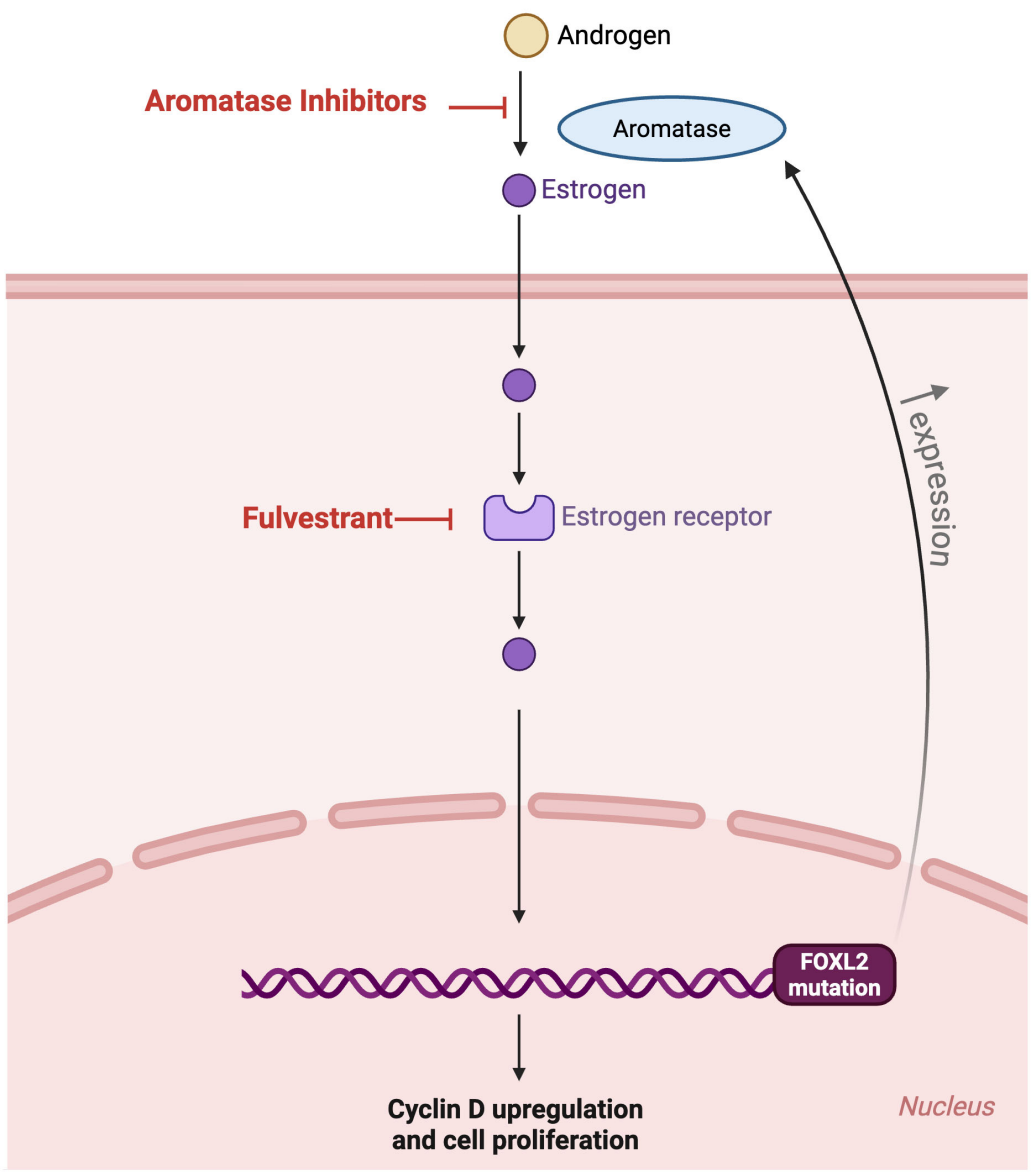
Proposed interaction between FOXL2 mutation, estrogen signaling, and cyclin D upregulation in adult granulosa cell tumors. The FOXL2 mutation may promote aromatase expression, thereby increase estrogen production and activating estrogen receptor signaling. Estrogen receptor activation enhances transcription of proliferation-associated genes, including cyclin D isoforms, thereby contributing to activation of the cyclin D–CDK4/6–Rb pathway and cell proliferation. Aromatase inhibitors suppress estrogen synthesis, whereas fulvestrant inhibits estrogen receptor signaling. CDK, cyclin-dependent kinase; ER, estrogen receptor; FOXL2, forkhead box L2; Rb, retinoblastoma protein.

Overall, endocrine therapy can provide meaningful disease control in a subset of patients with recurrent aGCT. Importantly, estrogen signaling promotes cyclin D expression and downstream CDK4/6 activation (as presented in **Figure 3**), providing a strong rationale for combining endocrine agents with CDK4/6 inhibitors (31).

## 5 Clinical evidence of CDK4/6 inhibition in adult granulosa cell tumors

Based on this biological rationale, early clinical studies have begun to evaluate CDK4/6 inhibitor–based strategies in recurrent aGCT. However, the currently available clinical evidence remains limited, consisting only of small case series and an ongoing prospective study (**Table 1**). Importantly, it relates almost exclusively to combinations of CDK4/6 inhibitors with endocrine therapy, with very limited data supporting CDK4/6 inhibitor monotherapy in aGCT.

**Table 1:** Summary of studies evaluating CDK4/6 inhibition in adult granulosa cell tumors.

Study	Study design	Population	Treatment	Key outcomes	Safety
Brodsky et al., 2025 (22)	Retrospective case series + preclinical models	11 pts with recurrent aGCT; median 5 lines of systemic therapies (range: 2-13) and 4 surgeries (range: 2-8)	Palbociclib in 9 pts (81.8%), abemaciclib in 2 pts (18.2%). 9 pts in combination with hormonal therapy and 2 pts alone.	PR in 3 pts (27.3%), SD in 6 pts (54.5%), PD in 2 pts (18.2%); median DOR 6 months	2 cases of dose reduction in palbociclib group (neutropenia) and 1 case in abemaciclib (diarrhea)
Albright et al., 2023 (32)	Retrospective case series	7 pts with recurrent aGCT aged 42–73 years; median 6 prior systemic therapies (range: 1-14), 3 surgeries (range:1-13), 3 pts received RTH	Palbociclib combined with hormonal therapy (letrozole in 6 pts; fulvestrant in 1 pt)	PR in 3 pts; SD in 2 pts; decline in inhibin A/B in 6 pts; median treatment duration and OS NR	3 cases of dose reduction; most common AE: neutropenia (5/7); fatigue and nausea (3/7); treatment discontinuation in 1 patient due to G3 fatigue.
Nanez et al., 2023 (conference abstract) (33)	Retrospective case series	4 pts with recurrent aGCT aged 30–73 years; 2–6 prior surgeries and 2–6 systemic therapies	CDK4/6 inhibitor combined with hormonal therapy (aromatase inhibitors, one patient fulvestrant)	PR in 1 pt; PD in 1 patient; mixed radiologic response with prolonged disease control in 2 pts; treatment duration 5–60 months	No AE reported
DiBernardo et al., 2025 (34)	Retrospective case series	4 pts with recurrent aGCT, aged 42.5 years (range: 27-73); median 5 prior recurrences (range: 3-9), 1 CTH, 2.5 hormonal therapies, 4.5 prior surgeries (range: 3-9), 2 pts received RTH	Palbociclib with hormonal therapy (aromatase inhibitors in 3 pts, fulvestrant in 1 pt)	Median treatment duration 38 months (range 6–60); 1 pt CR; 2 pts had responses >1 year before PD; 1 pt PD	Treatment well tolerated; no discontinuations due to toxicity
Ottenbourgs et al., 2024 (ALEPRO trial) (35, 36)	Phase II multicenter open-label clinical trial (ongoing)	Pts with recurrent ER–positive rare ovarian cancers including aGCT	Abemaciclib (150 mg BID) + letrozole (2.5 mg daily)	Primary endpoint: ORR (RECIST v1.1); target enrollment 40–100 pts; results pending	Safety monitoring included as secondary endpoint

AE, adverse event; Agct, adult granulosa cell tumor; BID, twice daily; CR, complete response; CTH, chemotherapy; DOR, duration of response; ER, estrogen receptor; G3, grade 3; NR, not reached; ORR, objective response rate; OS, overall survival; PD, progressive disease; PR, partial response; pts, patients; RECIST, Response Evaluation Criteria in Solid Tumors; RTH, radiotherapy; SD, stable disease.

The largest published experience is a retrospective case series of seven patients with recurrent aGCT treated with CDK4/6 inhibitors with hormonal therapy. Patients were heavily pretreated, with a median of 13 years since diagnosis (range 5–36). All received palbociclib, 6 with letrozole and 1 with fulvestrant. Three patients achieved a PR and two had SD. Six patients showed declines in inhibin A or B. At data cutoff, three patients remained on therapy, five were alive and median treatment duration and overall survival (OS) were not reached. Neutropenia was the most common adverse event (5/7 patients) and only one patient discontinued the treatment due to grade 3 fatigue (32).

A pilot case series of four patients with recurrent aGCT treated with a CDK4/6 inhibitor plus estrogen blockade (aromatase inhibitors in 3 patients and fulvestrant in 1 patient) reported additional clinical evidence. Treatment duration ranged from 5 to 60 months. One patient had a PR with decreased inhibin B and remained on therapy for 27 months. One had PD after five months, and two had mixed radiologic responses with prolonged disease control. Overall, three of four patients derived clinical benefit, and no treatment-related adverse events were reported (33).

Another institutional case series reported four patients with recurrent aGCT treated with CDK4/6 inhibitors plus aromatase inhibitors (n=3) or fulvestrant (n=1). Median age at diagnosis was 42.5 years, with a median of five prior recurrences. Median treatment duration was 38 months (range 6–60). At the last follow−up, one patient remained on therapy and had achieved CR, two had responses lasting over one year before progression and one had PD. Treatment was generally well tolerated, with no discontinuations for toxicity (34).

In the previously described study of Brodsky et al. (22) both preclinical and clinical data were reported. Among 11 heavily pretreated patients, disease control was achieved in 9 patients. Nine patients received palbociclib and two abemaciclib, including two treated with CDK4/6 inhibitor monotherapy. Dose reductions were required in three cases due to neutropenia or diarrhea, indicating a manageable safety profile.

A prospective clinical evaluation of CDK4/6 inhibition in this population is ongoing (NCT05872204). The ALEPRO trial is an international, multicenter, open-label phase II study of abemaciclib (150 mg twice daily) plus letrozole (2.5 mg daily) in patients with recurrent or metastatic ER–positive rare ovarian cancers, including aGCT. The primary endpoint is the ORR by the Response Evaluation Criteria in Solid Tumors (RECIST) v1.1. The trial plans to enroll 40–100 patients, with recruitment completion expected in 2028 (35, 36).

Collectively, the interpretation of these findings is limited by the very small sample sizes, retrospective design, potential publication bias and heterogeneity in prior treatments and response assessment. Nevertheless, these reports suggest that the combination of CDK4/6 inhibitors with endocrine therapy may lead to disease stabilization or partial responses in a subset of patients with heavily pretreated recurrent aGCT.

## 6 Discussion

**Figure 4:**
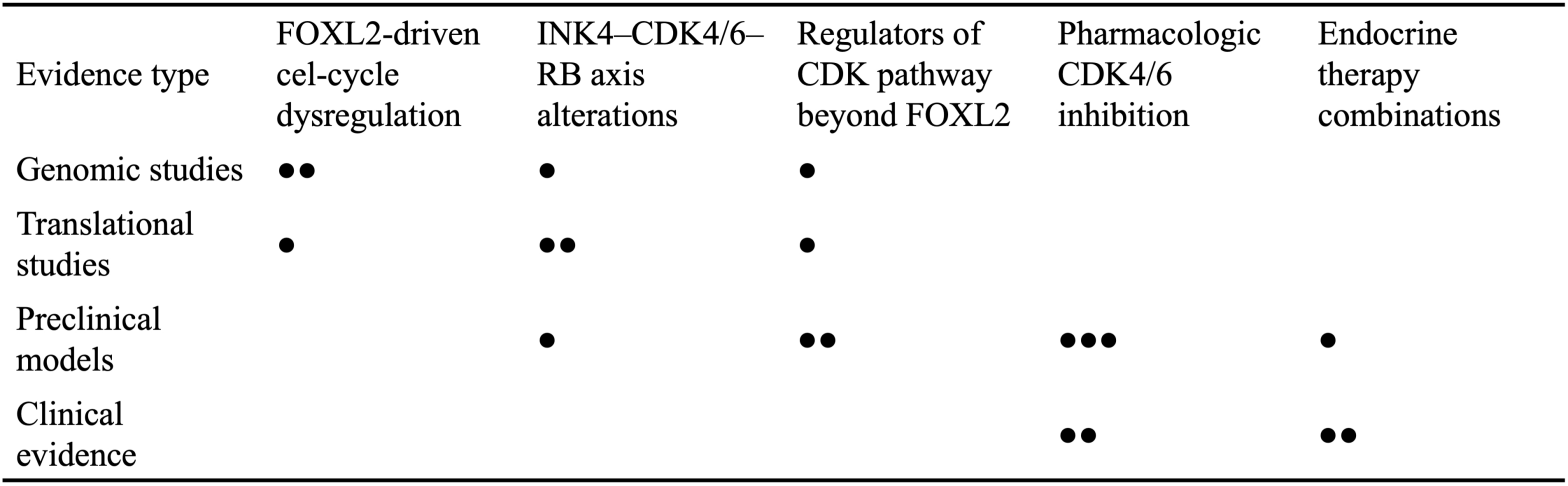
Evidence map of molecular and therapeutic evidence targeting the CDK4/6 pathway in adultgranulosa cell tumors. Dots represent the relative number of studies within each evidence domain. The map illustrates that most available data derive from genomic and mechanistic research, whereas direct clinical evidence evaluating CDK4/6 inhibitors remains limited. *FOXL2, forkhead box L2; INK4, inhibitors of cyclin-dependent kinase 4; CDK4/6, cyclin-dependent kinases 4 and 6; RB, retinoblastoma protein.*.

This scoping review highlights convergent translational, preclinical and early clinical evidence supporting the cyclin D–CDK4/6–Rb axis as a potential therapeutic target in aGCT. Molecular studies consistently show dysregulated cell-cycle control, including alterations in INK4 inhibitors, Rb pathway components and proliferation-related genes, while functional data suggest that regulators such as RUNX3 may further enhance CDK4/6 pathway activation. Preclinical models, including cell lines, organoids and xenografts, demonstrate that pharmacologic CDK4/6 inhibition can suppress tumor growth. However, clinical evidence remains limited to small retrospective case series, using CDK4/6 inhibitors in combination with endocrine therapy and an ongoing phase II trial may provide the first prospective evaluation of this strategy. The distribution of available evidence across translational, preclinical and clinical domains is summarized in **Figure 4**.

Experience from other malignancies provides additional context for the potential role of CDK4/6 inhibition in aGCT. In hormone receptor–positive breast cancer, CDK4/6 inhibitors combined with endocrine therapy have become a standard treatment and significantly improve PFS and OS in both palliative and curative settings, establishing cell-cycle targeting as an effective therapeutic strategy in tumors driven by hormonal signaling (37). Beyond breast cancer, CDK4/6 inhibitors have also demonstrated antitumor activity in several other solid malignancies characterized by dysregulated cell-cycle control, including ovarian cancer, liposarcoma, melanoma and head and neck squamous cell carcinoma (38). In a phase II study evaluating palbociclib in patients with recurrent ovarian cancer, clinically meaningful disease stabilization was observed in a subset of patients with low-grade tumors (39). A potential explanation for the observed activity of CDK4/6 inhibitors in certain ovarian tumors may lie in the biological characteristics shared by several low-grade malignancies. Tumors with relatively indolent growth kinetics and preserved dependence on hormone signaling pathways may remain particularly reliant on cyclin-D–CDK4/6–Rb–mediated cell-cycle progression. In this context, aGCT shares important biological similarities with other low-grade ovarian neoplasms, including hormone receptor expression and slower proliferative dynamics, which may render them especially susceptible to therapeutic strategies targeting cell-cycle regulation (31, 38).

Interestingly, endocrine treatment strategies reported in aGCT target multiple levels of estrogen signaling, including hypothalamic–pituitary regulation (GnRHa), estrogen synthesis (aromatase inhibitors) and estrogen receptor signaling (tamoxifen or fulvestrant). This likely reflects the complex hormonal biology of aGCT and suggests that simultaneous targeting of complementary components of estrogen-driven signaling may enhance antitumor activity. Given the close interaction between estrogen receptor activation and cyclin D–CDK4/6–Rb pathway signaling, combinatorial approaches integrating endocrine therapy with CDK4/6 inhibition may provide more effective suppression of proliferative signaling than either strategy alone (9, 12).

Inhibin, particularly inhibin B, is an established biomarker in aGCT and may reflect disease burden and treatment response. In a large single-center cohort, inhibin B demonstrated higher sensitivity for detection of granulosa cell tumors than inhibin A (89% vs. 67%), while both markers showed 100% specificity (40). Several reports included in this review also described decreases in inhibin levels during endocrine therapy, suggesting that biochemical response may parallel clinical benefit in selected patients.

At the same time, both intrinsic and acquired resistance to CDK4/6 inhibitors represent important challenges that may limit long-term therapeutic benefit. Several mechanisms have been described, including loss or functional inactivation of the Rb protein, amplification of cyclin E1 (CCNE1), activation of compensatory signaling pathways such as phosphatidylinositol 3-kinase/protein kinase B/mechanistic target of rapamycin (PI3K/AKT/mTOR) and adaptive changes in cell-cycle regulators that bypass CDK4/6 dependency (41, 42). These findings rationalize combination strategies to enhance treatment efficacy and delay resistance.

Future research should focus on prospective evaluation of CDK4/6 inhibitor–based strategies in aGCT, particularly in combination with endocrine therapies targeting complementary components of estrogen signaling. Biomarker-driven studies integrating genomic and transcriptomic profiling may help identify predictors of response and refine patient selection. Improved preclinical models, including genetically engineered and patient-derived systems, may further facilitate the investigation of resistance mechanisms and rational combinatorial strategies. Ultimately, a deeper understanding of the molecular drivers of aGCT progression and treatment resistance will be essential for translating promising preclinical findings into effective targeted therapies for patients with this uncommon ovarian malignancy. Future research should also focus on identifying the optimal endocrine partner for CDK4/6 inhibition in aGCT, as clinical benefit may depend not only on the CDK4/6 inhibitor itself but also on the specific hormonal backbone.

Several limitations should be acknowledged. First, this scoping review aimed to map the available evidence rather than to perform a formal systematic review with quantitative synthesis; therefore, the methodological quality and risk of bias of included studies were not formally assessed. Second, the currently available clinical evidence regarding CDK4/6 inhibition in aGCT is limited and consists mainly of small retrospective case series and conference reports, which may introduce reporting and publication bias. Third, due to the rarity of this tumor type, the number of eligible studies remains small and heterogeneous in terms of study design and patient populations. Although a comprehensive search strategy was applied, evidence on endocrine combination strategies was synthesized narratively rather than through a dedicated systematic search, which may have limited the completeness of this aspect of the review. Finally, the small size of the retrospective series and the possibility of overlapping patient populations across reports from collaborating tertiary centers may introduce a risk of inadvertent double-counting, a recognized limitation in rare-tumor reviews.

## 7 Conclusions

CDK4/6 inhibition represents a biologically justified therapeutic strategy in aGCT, supported by convergent translational, preclinical and early clinical evidence. Although the available clinical data remain limited, the observed signals of activity—particularly in combination with endocrine therapy—support further prospective evaluation. Overall, these findings support CDK4/6 inhibition as a biologically plausible therapeutic approach that merits further prospective clinical evaluation.
